# Paternal cytokine administration alters sperm small ncRNAs and offspring brain and behavior

**DOI:** 10.1038/s41380-026-03674-5

**Published:** 2026-06-19

**Authors:** Pamudika Kiridena, Nicholas van de Garde, Bethany A. Masson, Carolina Gubert, Sonali N. Reisinger, Anthony J. Hannan

**Affiliations:** 1 Florey Institute of Neuroscience and Mental Health, Parkville, VIC Australia; 2 Florey Department of Neuroscience and Mental Health, University of Melbourne, Parkville, VIC Australia; 3 Department of Biochemistry & Chemistry, School of Agriculture, Biomedicine and Environment, La Trobe University, Bundoora, Australia; 4 Department of Anatomy and Physiology, University of Melbourne, Parkville, VIC Australia

**Keywords:** Neuroscience, Schizophrenia, Predictive markers, Molecular biology, Biochemistry

## Abstract

Paternal pre-conceptual exposure to pathogenic infection can alter offspring phenotypes via changes to sperm epigenetics, including small non-coding RNAs (ncRNAs). This phenomenon occurs even in the absence of infection when pathogen mimetics trigger paternal immune activation (PIA). While this implicates a shared component of the immune response, the specific mechanisms responsible remain unknown. Cytokines are a key component of innate immunity and are highly upregulated following exposure to both pathogens and their mimetics. Here we investigate whether paternal administration of the pro-inflammatory cytokines interleukin-1β (IL-1β) and tumor necrosis factor-α (TNF-α) recapitulates alterations to sperm small ncRNAs and offspring phenotypic changes from other models of PIA. C57BL/6J male mice were administered a single dose of IL-1β, TNF-α, or vehicle, prior to mating with naïve female mice, to produce offspring. Offspring from IL-1β-treated fathers exhibited altered fasting responses and female-specific alterations to stress-coping behavior. In response to paternal TNF-α, male offspring showed increased anxiety-like behavior. Analysis of paternal sperm small ncRNA revealed that IL-1β significantly downregulated several transfer RNA-derived small RNAs (tsRNAs) and P-element-induced wimpy testis (PIWI)-interacting RNA (piRNA) clusters. Surprisingly, paternal TNF-α only slightly altered small ncRNAs, downregulating a single piRNA cluster. These results partially recapitulate offspring phenotypic changes following paternal exposure to severe acute respiratory syndrome coronavirus 2 (SARS-CoV-2, in a COVID-19 mouse model) and polyinosinic:polycytidylic acid (poly I:C, a viral mimetic). Ultimately, we provide the first evidence that cytokines have intergenerational effects on brain and behavior, and that they contribute to altered offspring phenotypes in viral-like PIA. This study demonstrates that paternal cytokines are causally involved in a mechanism of epigenetic inheritance following PIA, and highlights the importance of PIA as a pre-conceptual factor that may modulate offspring health, including risk of neuropsychiatric disorders.

## Introduction

Psychiatric disorders arise from the complex interplay of genetic and environmental factors which shape neurobiological processes. Limited understanding of this multifactorial aetiology has long posed a barrier in the development of effective strategies for prevention, early intervention, and treatment. Epigenetic inheritance, a facet of gene-environment interactions, offers increasing insights into the risk of metabolic diseases [1–3], cancers [4–6], psychiatric disorders [7–9], and other diseases in humans. Supporting this notion, the parental environment has been shown to alter offspring physiology and behavior in rodent models; however, most studies have focused on the maternal line [9]. Research now highlights a parallel role for paternal epigenetic inheritance, with influences such as paternal toxins [10–12], stress [13–16], diet [17–20], exercise [21, 22], gut microbiome [23, 24], and immune activation [25–29] altering offspring phenotypes. This can occur via modifications to the sperm epigenome including deoxyribonucleic acid (DNA) methylation, histone modifications, and altered small and long non-coding RNA (ncRNA), which are delivered to the oocyte upon fertilization and exert effects on early development [30–33].

Epigenetic modifications in the context of immune activation have gained special attention in recent years, driven by the Coronavirus Disease 2019 (COVID-19) pandemic and heightened awareness of the threat of future infectious disease outbreaks [34]. Increasing evidence suggests that paternal immune activation (PIA) prior to conception alters offspring phenotypes via epigenetic mechanisms [25–29, 35, 36]. Preconceptual infection with *Toxoplasma gondii* in mice has been shown to induce sex-dependent cognitive, anxiety-like, and depression-like behavioral changes in offspring (intergenerational effects) and grand offspring (transgenerational effects) [25]. More recently it has been demonstrated that paternal infection with Severe Acute Respiratory Syndrome Coronavirus 2 (SARS-CoV-2) can alter offspring anxiety-like behavior and fasting responses, with limited impacts on grand offspring [28]. In these studies, offspring phenotypic changes have been attributed to epigenetic mechanisms, evidenced by alterations to paternal sperm small ncRNAs and the partial recapitulation of offspring phenotypes following microinjection of sperm RNA into fertilized oocytes. The small ncRNA profile of mature sperm is generally comprised of high proportions of transfer RNA (tRNA)-derived small RNAs (tsRNAs) and ribosomal RNA-derived small RNAs (rsRNAs) along with microRNA (miRNA) and P-element-induced wimpy testis (PIWI)-interacting RNA (piRNA) [37–39]. The composition of the small ncRNA population shifts dramatically during epididymal transit when epididymosomes deliver small RNA to the maturing sperm [38, 40, 41]. Notably, the RNA cargo of epididymosomes can be altered in response to paternal environment, offering a potential mechanism through which offspring phenotypes may be altered [42]. Although the exact mechanisms through which parasitic and viral infection alter sperm small ncRNA and subsequent offspring phenotypes are unclear, studies have suggested this phenomenon is not due to the infection itself, but rather a consequence of the immune response.

Even in the absence of infection, PIA via pathogen mimetics can alter offspring phenotypes. Intraperitoneal (IP) administration of the viral mimetic polyinosinic:polycytidylic acid (poly I:C) in fathers prior to conception has been shown to alter sperm small ncRNA profiles and increase offspring depression-like behavior and alter offspring physiology including brain mass, immune response, and fasting responses [26]. Similarly, paternal pre-conceptual exposure to the bacterial mimetic lipopolysaccharide (LPS) can alter offspring behavior via alterations to sperm small ncRNA, however there are differential effects, with decreased anxiety, heightened sociability, and increased cognitive function [27]. In both pathogen infection mimetic models, limited impacts are observed in grand offspring, mirroring the effects of paternal SARS-CoV-2 [28]. These studies suggest that PIA without infection is sufficient to alter paternal sperm small ncRNA and offspring phenotypes, particularly anxiety-like and depression-like behaviors. As the directionality of effects differ with the source of PIA, the precise nature and dynamics of the immune challenge, and subsequent immune response, may determine offspring phenotypic changes.

While poly I:C engages the toll-like receptor 3, retinoic acid inducible gene 1 protein, and melanoma differentiation-associated protein 5, LPS activates toll-like receptor 4. This leads to the activation of an overlapping but distinct combination of adaptor proteins, transcription factors, and pro-inflammatory genes [43–45]. This includes the upregulation of several key pro-inflammatory cytokines such as interleukin-6 (IL-6), interleukin-1β (IL-1β), and tumor necrosis factor-α (TNF-α also known as TNF) [43–45] which are produced by a variety of cell types, particularly macrophages, monocytes, and dendritic cells [46–48]. These cytokines play crucial roles in driving sickness responses such as fever, weight loss, and diminished food and water intake [49–53] which are shared features of the immune response against pathogens and their mimetics [44, 45]. Consequently, it is possible that similarities in cytokine profiles are responsible for overlapping offspring phenotypes in PIA models, while variations in cytokine profiles drive distinct offspring phenotypes.

Cytokines also play important roles in male reproduction, modulating testicular function and spermatogenesis [54, 55]. Previous research has indicated that the blood-testis barrier (BTB) is disrupted during viral and bacterial infections, with cytokines being implicated in this process [56–62]. Although the role of cytokines has not yet been studied in the context of PIA, several studies have highlighted a role for cytokines in maternal immune activation (MIA), where female mice experience systemic inflammation via pathogenic or non-pathogenic means during pregnancy. In rodent models, administration of a single dose of cytokines in pregnant mothers alters offspring neurodevelopment and behavior [63–68]. For instance, when administered IL-6, offspring exhibit deficits in pre-pulse inhibition, latent inhibition, and socialisation [68]. Interestingly, coadministration of anti-IL-6 antibody alongside poly I:C can rescue these offspring phenotypes [68]. These findings suggest that IL-6 may be driving the effects of offspring phenotypic changes in MIA models, however it is unclear whether these changes occur via epigenetic mechanisms, placental mechanisms, or other indirect or direct effects on the developing fetal brain [69].

Cytokines are a key component of the innate immune response and, given their involvement in male reproductive processes, BTB integrity and altering offspring health in MIA models, they represent a mechanism worthy of further exploration in the context of PIA. Elevated blood cytokine levels during PIA could alter the sperm epigenome and subsequent offspring phenotypes and, by elucidating the role of cytokines in this process, we can better understand the mechanisms driving paternal epigenetic inheritance in PIA models. We hypothesized that exogenous cytokine administration would elicit paternal sickness responses, modify the sperm epigenome, and alter offspring phenotype in a manner which recapitulates outcomes from other PIA models. Here, we show that paternal cytokine administration of IL-1β and TNF-α elicits a robust sickness response and is sufficient to alter sperm epigenetics and offspring phenotypes in a way which partially recapitulates PIA via SARS-CoV-2 infection and poly I:C immune activation. This study provides the first evidence of how paternal cytokines alter offspring phenotypes and builds understanding of how PIA during pre-conception is contributing to offspring health and risk of neuropsychiatric disorders.

## Materials and methods

### Animals

C57BL/6J mice were acquired from Ozgene Animal Research Centre (Ozgene ARC; Perth, Australia) between 6–8 weeks of age. Upon arrival, F0 male mice were housed in pairs while F0 female mice were housed in groups of 4–6 per box. F1 offspring were generated from F0 mice in-house, as described below. At weaning, F1 offspring litters were housed in groups of 4–6 per box, separated by sex and paternal treatment. All mice were kept in open-top housing with standard wood-chip bedding (Tapvei Estonia Oü; Harjumaa, Estonia), soft paper tissues, and red houses with *ad libitum* access to standard chow (Ridley; Melbourne, Australia) and water. Rooms were temperature and humidity-controlled (22 °C ± 2, 45%) on a 12 h light/dark cycle with lights on at 0800 h. All experiments were conducted according to the National Health and Medical Research Council (NHMRC) research guidelines and regulations and with approval from the Florey Institute of Neuroscience and Mental Health Animal Ethics Committee (#23-013-FINMH).

### Cytokine administration

Recombinant murine IL-1β (Cat. #211-11B-1MG), TNF-α (Cat. #315-01A-1MG), and IL-6 (Cat. #216-16-1MG) were purchased from PeproTech (New Jersey, United States) and prepared as per the manufacturer’s instructions. Cytokines were diluted in a sterile solution of phosphate-buffered saline (PBS) and 0.1% bovine serum albumin (BSA) (CAS #9048-46-8) from Sigma-Aldrich (Missouri, United States). Following a 2-week habituation period, 8–9-week-old C57BL/6J male mice received an intraperitoneal (IP) injection of key pro-inflammatory cytokines. When determining the contribution of individual cytokines to the sickness response, mice were administered cytokines in three separate cohorts: IL-1β (50 μg/kg, *n =* 8 per group) or TNF-α (250 μg/kg, *n =* 8 per group) alone; IL-6 (250 μg/kg, n = 8 per group) alone; or IL-6 (250 μg/kg) + IL-1β (0.5 μg/kg) in combination (*n =* 10 per group). For these initial studies, rectal body temperature was measured 2 h post-injection (PI) and every 24 h thereafter for 6 days. The addition of 0.1% BSA had no significant impacts on sickness responses  (Supplementary Figure 1a–d) and was therefore used in all further studies. For F0 male mice, IL-1β (50 μg/kg) or vehicle, or TNF-α (250 μg/kg) or vehicle (*n =* 10 per group) was administered IP in two separate cohorts. In all experiments, food, water, and body weight were measured 24 h and 2 h before cytokine administration and every 24 h thereafter for 6 days. Locomotor activity was measured 4 h PI in a 15 min open field test. The arena consisted of a transparent Plexiglas box (ENV-510, 27 cm × 27 cm × 20.5 cm, Med Associates Inc.; Vermont, United States) and infrared beam arrays (ENV-256, Med Associates Inc.). Automated activity monitoring software was used to determine distance travelled. Mice were excluded from the analysis if they escaped the testing arena (*n =* 1 mouse excluded from the initial TNF-α cohort).

### Breeding

Prior to a full spermatogenesis cycle [70], approximately 4 weeks after injection, cytokine-treated F0 male mice were mated with age-matched naïve F0 female mice. After a mating period of 5 days, male mice were sacrificed for tissue collection. Female mice were single-housed and red plastic enclosures removed to allow for unobstructed maternal care observations. Following the birth of a litter, on postnatal day (PND) 0, litter size was recorded. From PND 1–7 maternal care was recorded once every morning and afternoon as described below. Following the conclusion of maternal care evaluation, the sex of pups was determined between PND 8–10. F1 mice were weaned between PND 24–27, and litters were mixed using random number generators, separated by sex and paternal treatment. F1 mice were group housed with 4–6 per box.

### Maternal care

Maternal care was scored daily from PND 1–7 over two sessions per day (0830 – 1000 and 1630 – 1800) for each litter in the IL-1β control (*n =* 4), IL-1β treatment (*n =* 6), TNF-α control (*n =* 9), and TNF-α treatment (*n =* 9). Behavior was observed via instantaneous scan sampling to assess the quality and quantity of maternal care as previously described [23, 26]. In each session, observations were made 30 times per litter, approximately once every 90 s. The presence and absence of self-maintenance (self-grooming, eating/drinking), nurturing (nest building, arch-backed nursing, passive nursing, grooming/licking pups) and neglectful (climbing, digging, pup outside nest) behavior were recorded. Observations were conducted by a single scorer blinded to group assignment. The relative frequency of these behaviors was calculated by averaging the proportion of each behavior across the two sessions.

### Behavior

F1 male control (IL-1β: *n =* 13, TNF-α: *n =* 14), male treatment (IL-1β: *n =* 13, TNF-α: *n =* 14), female control (IL-1β: *n =* 13, TNF-α: *n =* 14), and female treatment (IL-1β: *n =* 14, TNF-α: *n =* 15) mice began behavioral testing upon reaching adulthood, at 8 weeks of age. Behavioral tests were carried out from the least to most stressful in the following order: large open field (LOF); elevated plus maze (EPM); novelty-supressed feeding test (NSFT); and Porsolt swim test (PST). The saccharin preference test (SPT) was conducted last due to the requirement of individual housing. Mice were given at least 24 h of rest between individual behavioral tests with a 48 h resting period for more stressful tests such as NSFT and PST. All testing was carried out between 0800 and 1700 under 30–50 lx unless specified otherwise. Testing for male and female mice were conducted on different days where possible, with experimenters blinded to experimental groups. Before beginning any behavioral test, mice were acclimatized to the room for at least 1 h, unless specified otherwise. Between trials, arenas were cleaned with 20% ethanol for all behavioral tests.

### Large open field

The LOF was performed to evaluate anxiety-like behavior and locomotion as previously described [71]. Mice were placed in the corner of the arena (opaque PVC box, 100 cm x 100 cm x 20 cm), which was illuminated at 1000 lx (using two flood lights placed on the corners of the arena) in the centre, and allowed to explore for a 10 min period. For analysis, the arena was divided into a centre zone (50% of total arena) and outer zone (50% of total arena). The total distance travelled over 10 min, latency to centre, and the percentage of total time spent in the centre zone were automatically calculated using an overhead camera and TopScan Lite Software (CleverSys Inc.; Virginia, United States).

### Elevated-plus maze

The EPM was used to evaluate anxiety-like behavior as described previously [26]. Mice were placed in an arena elevated approximately 40 cm off the ground. The arena consisted of two open arms (5 cm x 30 cm), two closed arms (5 cm x 30 cm x 15 cm opaque walls), and a central zone (5 cm x 5 cm) which intersect at 90 degrees to form a plus sign. Mice were placed in the centre zone facing the open arms and allowed to explore all arms for 5 min. Total distance travelled, and time spent (%) in the open and closed arms was automatically calculated using an overhead camera and TopScan Lite Software (CleverSys Inc.).

### Social interaction test

The SIT was used to assess social interaction abilities as previously described [26]. Sociability was assessed over three trials in a transparent Plexiglas box (San Diego Instruments, Inc.; California, United States; 40 cm x 37.5 cm x 10 cm) divided into 3-chambers consisting of a central chamber connected to two outer chambers (16 cm x 37.5 cm x 10 cm), each with a metal cage at the end. In the first trial, the test mouse was placed in the central chamber and habituated to the arena for 5 min. The mouse was then removed from the arena. In the second trial a “guest” mouse, sex and age matched, was placed in a cage in either the left or right chamber with the test mouse being placed in the central chamber. The test mouse was allowed to explore for 10 min. Between each trial, the arena was cleaned with 20% ethanol. The experimental mouse was tracked automatically using an overhead camera and TopScan Lite Software (CleverSys Inc.).

### Novelty-suppressed feeding test

The NSFT was used to assess the anxiety-like behavior hyponeophagia as previously described [26]. Mice were fasted for 24 h with water available *ad libitum*. Mice were weighed pre- and post-fast to determine bodyweight loss after 24 h. During testing, mice were placed in a brightly lit (750 lx) novel arena (60 cm x 60 cm x 60 cm) filled with 3 cm of bedding and a standard chow pellet in the centre affixed to a circular piece of filter paper. The latency to take the first bite from a chow pellet was recorded, with the trial ending after 10 min. Following testing, the mouse was placed alone in a new cage containing a pre-weighed food pellet and allowed to eat for 5 min. The amount of food consumed was recorded. Bedding was changed on each testing day and between sexes, with fresh bedding mixed with bedding from each cage to be tested.

### Porsolt swim test

The PST was used to evaluate stress-coping behavior [72, 73] as previously described [23]. Mice were individually placed in a 2000 mL glass beaker partially filled with approximately 1400 mL water at 24 ± 1 °C. Mice were recorded for 6 min using digital cameras. Mice were dried and warmed with towels and heat lamps prior to being returned to a holding cage or home cage. The video footage was analyzed manually to determine latency to first immobility episode by a blinded experimenter, and the final 4 min of the test were used to automatically determine total time immobile via the DepressionScan software suite (CleverSys Inc.).

### Saccharin preference test

The SPT was used as a measure of anhedonia, a depression-like behavior, and was conducted as previously described [23]. Mice were single housed with *ad libitum* access to food and water. In the first 24 h, mice were given water in two identical 50 mL falcon tubes with sippers to allow for habituation to the two-bottle setup. For the first testing night, mice were given one tube containing approximately 40 mL of 0.1% saccharin solution (#S1002, Sigma-Aldrich) and the other containing 40 mL of standard drinking water with placement on the left or right side being randomized. The placement of the two bottles was reversed for the second night of testing to control for side preferences. The weight of solution consumed was recorded on the habituation day and the first and second testing nights by a blinded experimenter. Saccharin preference index (%) was determined by calculating the amount of saccharin consumed in comparison to total fluid consumed over a 24 h period.

### Tissue collection

F1 male and female mice were administered a lethal dose (80 mg/kg) of pentobarbitone sodium (Lethabarb) (Virbac; NSW, Australia) via IP injection followed by cervical dislocation. For collection of fresh whole brains, the head was decapitated and the scalp removed to expose the skull. Small dissection scissors were used to make a midsagittal incision in the skull, taking caution to avoid pressure on the brain. Forceps were used to remove pieces of the skull. After inverting the head, the trigeminal and optic nerves were severed using a spatula, allowing the brain to be released and extracted. Whole brain mass was recorded prior to dissections of the prefrontal cortex, striatum, hippocampus, and hypothalamus. This whole brain collection was carried out on a subset of F1 offspring with experimenters blinded to group condition.

### Epididymal sperm collection

F0 male mice were administered a lethal dose (80 mg/kg) of pentobarbitone sodium (Virbac) via IP administration followed by cervical dislocation. Caudae epididymides and vasa deferentia were separated and cleaned from the epididymal fat pad as previously described [26]. Caudae epididymides contain mature sperm which are responsible for fertilizing the ova and therefore are more relevant to any alterations in offspring phenotype. Consequently, caudae epididymides were bisected and immersed in tubes containing 1 mL of Modified Tyrode’s Medium 6 (MT6) [26] (solution contains (in mM): 124 NaCl, 2.68 KCl, 17.14 CaCl_2_, 3.2 NaH_2_PO_4_·H_2_O, 0.49 MgCl_2_·6H_2_O, 25 NaHCO_3_, 5.6 D-glucose, 4 mg/mL BSA, 28.2 Phenol Red) kept at 37 °C in and saturated with CO_2_ overnight. Tubes containing the epididymides in MT6 were incubated in a heating block at 37 °C for a minimum of 30 min. Tissues were removed and sperm was pelleted by centrifugation at 400 g for 10 min. Supernatant was removed, and sperm pellets were stored at −80 °C.

### Small RNA extraction

Prior to total RNA extraction, all samples underwent somatic cell lysis to remove contamination of samples by somatic cells, which may skew sequencing results. 1 mL of somatic cell lysis buffer [41] (solution contains 0.01% sodium dodecyl sulfate, 0.005% Triton X-100 in nuclease-free water) was added to each frozen sample and homogenized. Samples were incubated on ice for 15 min followed by centrifugation at 3000 g for 4 min at 4 °C. The supernatant was removed and washed twice with PBS. Sperm lysis was carried out in QIAzol (Cat. #79306, QIAGEN; Hilden, Germany). Total RNA was extracted and purified from sperm samples as previously described [26]. The QIAGEN miRNeasy Mini Kit (Cat. #217004) and RNase-Free DNase Set (Cat. #79254) was used according to the manufacturer’s instructions to extract total RNA and carry out DNase treatment respectively. The RWT buffer was prepared with isopropanol. For the IL-1β cohort, each condition contained 4 biological replicates and for the TNF-α cohort there were 5 biological replicates, each containing sperm RNA pooled from 2 male mice to ensure adequate RNA concentration for sequencing (following the addition of ethanol for precipitation). The final elution step was repeated three times with 20 μl of RNase-free water. RNA was quantified using the Qubit RNA Broad Range Assay Kit (Cat. # Q10210; Thermo Fisher Scientific; Massachusetts, United States) in a Qubit 4 Fluorometer (Thermo Fisher Scientific). Quality control was carried out by the 4200 TapeStation System from Agilent (California, United States) using the RNA ScreenTape and Buffer (Cat. #5067-5576, 5067-5577) and RNA ScreenTape Ladder (Cat. #5067-5578) to determine RNA quality and potential somatic cell contamination.

### Small RNA sequencing

The standard NEXTflex Small RNA v4 library was prepared with bead size selection for Illumina Next-Generation Sequencing using the NovaSeq X 10B lane, 300-cycle at the Australian Genome Research Facility. All samples were processed on a single flow cell lane with 150-bp-paired-end reads. An average of 24 100 840 reads per biological replicate were obtained for the IL-1β cohort and 24 602 294 for the TNF-α cohort.

### Small ncRNA annotation and analyses

Small RNA sequence annotation was carried out using SPORTS v1.1.2, a non-coding RNA annotation and analysis software optimized for rsRNA- and tRNA-derived small RNAs [74]. Adapter removal and trimming (15–45 bp inclusive) was conducted in SPORTS v1.1.2. Read quality was assessed using FastQC [75] to verify clean reads. Following quality control, there were 14 960 005 and 14 282 763 clean reads per biological replicate across the IL-1β and TNF-α cohorts, respectively. SPORTS v1.1.2 was used to map reads to the following databases sequentially: (1) miRbase v22.1 for miRNA; (2) National Center for Biotechnology Information (NCBI) for ribosomal RNA (3) GtRNAdb for tRNA; (4) ensemble and Rfam for other ncRNA; and (6) piRBase and piRNABank for piRNA. SPORTS v1.1.2 default databases were used except for miRbase v22.1, where the database was compiled from mature.fa files pre-processed according to SPORTS v1.1.2 documentation. Approximately 91.39% of clean reads in the IL-1β cohort and 93.11% in the TNF-α cohort to aligned to these databases and/or mouse reference genome (GRCm38). Output was used to calculate reads per million and restructured into a matrix formatted for differential expression analysis in DEseq2 [76]. Here, piRNAs were removed due to inclusion in a separate pipeline for piRNA cluster analysis. In DEseq2 the false discovery rate (FDR) was set to 5% using the Benjamini and Hochberg method with independent filtering in DEseq2 removing low count genes. Graphs were generated using ggplot2 [77] package (v3.5.1), and heatmaps created using pheatmap [78] package (v1.0.12). Gene ontology (GO) analysis of differentially expressed tsRNAs was carried out by taking the seed sequence (positions 2–8 inclusive of the 5’ end of the molecule) and searching in TargetScan [79] (https://www.targetscan.org/vert_50/seedmatch.html) to identify messenger RNA (mRNA) targets. Target gene names were entered into the Gene Expression Database [80] (https://www.informatics.jax.org/gxd/batchSearch) from Mouse Genome Informatics to filter for genes that are only expressed during early embryonic development up until day 5 (Theiler Stage 1–7 inclusive). Following filtering, 1 141 genes remained from the initial 1 236 target genes. Functional annotation was carried out using Enrichr to identify cellular, molecular, biological, and knock-out phenotype ontology. Knock-out phenotypes are determined from data collected by the Knockout Mouse Phenotyping Program (KOMP2) which draws on the results from 120 peer-reviewed publications to characterize the function of protein coding genes.

For piRNA cluster analysis, reads were collapsed into unique sequences to remove redundancies and allow for efficient mapping. The bowtie 2 [81] alignment tool was used to map collapsed reads to the mouse reference genome [82] (GRCm38, GENCODE https://www.gencodegenes.org/mouse/release_M25.htmL). On average 75.57% of reads per sample were successfully mapped in the IL-1β cohort and 76.79% of reads per sample in the TNF-α cohort. The SAM files generated by bowtie 2 were used in proTRAC (v2.4.3), a piRNA cluster prediction tool. Default settings were used except for 1TorA which was set to 0. This parameter normally adjusts for 1U and 10 A biases caused during the ping-pong cycle, however male gametes are mostly comprised of RapiRNAs, which rarely undergo ping-pong amplification mechanisms during spermiogenesis [83]. piRNA length was set to 21–33 [83]. From the generated list of piRNA clusters, the intersect gene annotation file (gencode.vM25.annotation, GENCODE) was used for identification of nearby genes. Bedtools [84] was used to generate a count matrix of the merged piRNA clusters which was later used for differential expression analysis. For differential expression analysis, read counts corresponding to each predicted piRNA cluster was generated. The DEseq2 package was used to identify statistically significant differentially expressed piRNA clusters. The false discovery rate (FDR) was set to 5% using the Benjamini and Hochberg method with independent filtering in DEseq2 removing low count genes. Circlize [85] was used to visualise piRNA cluster expression across samples.

### Statistical analyses

Statistical analyses and graphing were carried out in R (v4.4.2) using R Studio (v2023.06.01 build 764) and GraphPad Prism (v10.4.2) with data presented as mean ± standard error of the mean (SEM). For experiments investigating the contribution of cytokines to the sickness response, ANOVAs were used followed by Dunnet’s, Šídák’s, or Tukey’s test for multiple comparisons where applicable. For analysis of F1 behavioral data, linear mixed models (LMM) were used with paternal treatment and sex as fixed factors and litter as a random factor due to possible reductions in phenotypic variability from offspring being sired from the same father and mother. For repeated measures analysis, offspring ID was nested within litter. Data and residuals were visually assessed for linearity, normality, and homogeneity of variance. The Shapiro-Wilk test was used to assess for normal distribution and a general linear mixed model (GLMM) with gamma distribution used for latency data which deviated from the assumptions. Regression models were assessed using the emmeans package and the ‘joint_tests’ function. If paternal treatment or sex had a higher relative contribution, these contrasts were tested using the emmeans ‘pairs’ function. If the interaction term had a relatively equal or greater contribution than paternal treatment or sex, the ‘pairs’ function was used to determine the nature of this interaction with Tukey’s test where applicable. Sample sizes were chosen based on previous behavioral neuroscience research from our laboratory aiming to detect moderate to large effect sizes [23, 26–28]. Full statistics can be found in Supplement 1. The significance level used was α = 0.05.

## Results

### Paternal IL-1β and TNF-α administration in male mice induces a robust sickness response

**Fig. 1:**
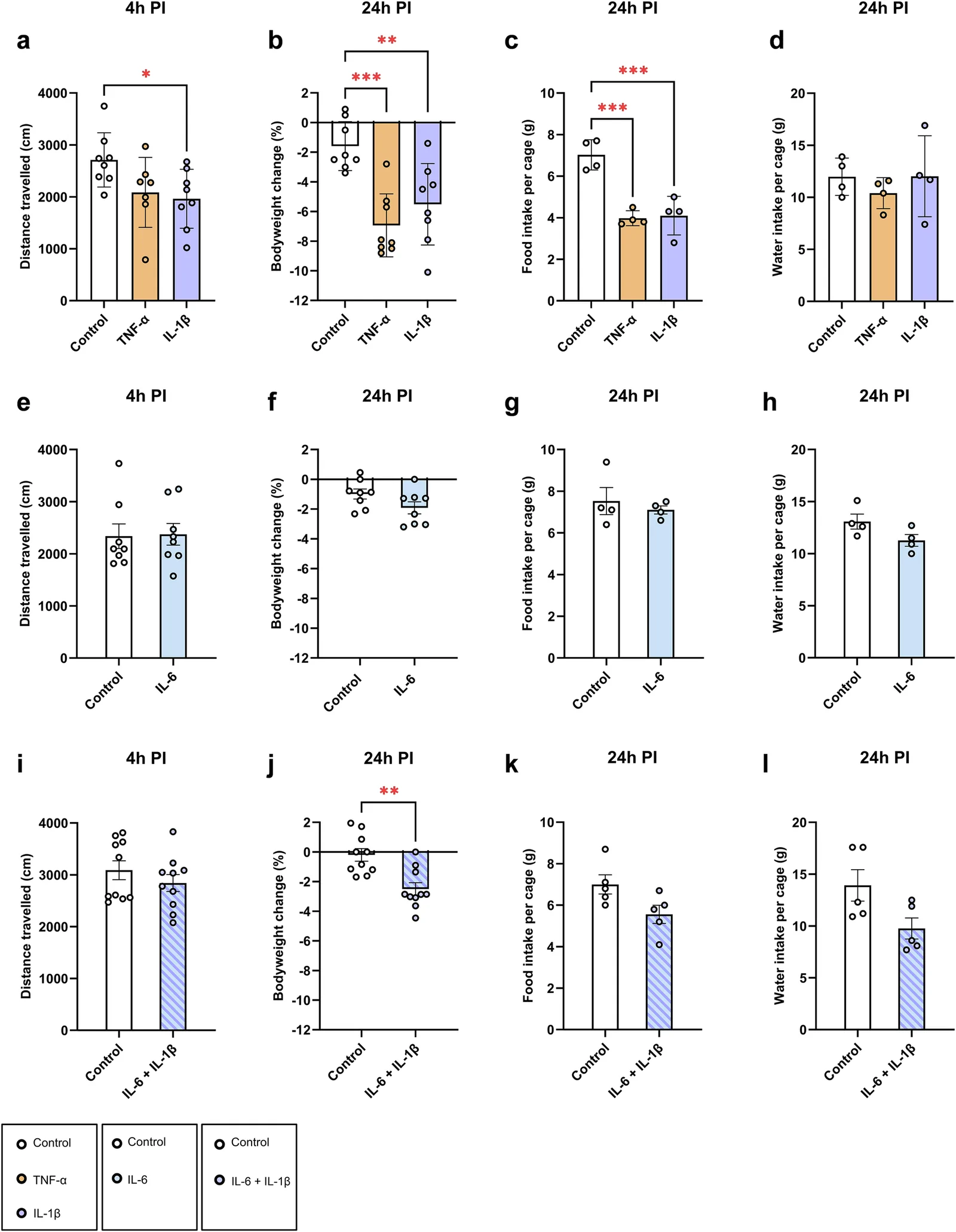
Sickness responses following a single dose of cytokines. Male mice received a single intraperitoneal (IP) injection of either IL-1β, TNF-α, IL-6, a combination of IL-6 + IL-1β, or vehicle, at 8-9 weeks of age. Sickness responses were measured. **(a)** Locomotor activity 4 h post injection (PI) in control, TNF-α, and IL-1β groups. **(b)** Bodyweight loss 24 h PI in control, TNF-α, and IL-1β groups. **(c)** Food intake 24 h PI in control, TNF-α, and IL-1β groups. **(d)** Water intake 24 h PI in control, TNF-α, and IL-1β groups. **(e)** Locomotor activity 4 h PI in control and IL-6 groups **(f)** Bodyweight 24 h PI in control and IL-6 groups. **(g)** Food intake 24 h PI in control and IL-6 groups**. (h)** Water intake 24 h PI in control and IL-6 groups**. (i)** Locomotor activity 4 h PI in control and IL-6 + IL-1β groups. **(j)** Bodyweight 24 h PI in control and IL-6 + IL-1β groups. **(k)** Food intake 24 h PI in control and IL-6 + IL-1β groups. **(l)** Water intake 24 h PI in control and IL-6 + IL-1β groups. Individual values and means are presented with error bars representing ± SEM (males *n* = 6–10 per group), *p < 0.05, **p < 0.01, ***p < 0.001.

To isolate which cytokines contribute to the shared sickness response observed across pathogenic and pathogen mimetic models of PIA, several pro-inflammatory cytokines were exogenously administered. Male mice received a single IP injection of either IL-1β, TNF-α, IL-6, IL-6 + IL-1β (combined cytokines), or vehicle solution, to determine whether they elicited a sickness response characterized by decreased locomotor activity, bodyweight loss, reduced food and water intake, or fever. Cytokine doses were chosen based on previous literature where sickness responses were reported at 50 μg/kg for IL-1β [52, 86], 250 μg/kg for TNF-α [52, 87] and 250 μg/kg for IL-6 [68]. There was a significant treatment effect on locomotor activity (*P =* 0.0428) with *post hoc* analysis revealing that only IL-1β administration led to a statistically significant decrease in distance travelled when compared to controls (IL-1β: P_adj_ = 0.0350; TNF-α: P_adj_ = 0.0942, Fig. 1a). Baseline bodyweights did not differ across the groups (*P =* 0.407 Supplementary Figure 1e) and as expected, there was a statistically significant difference in bodyweight 24 h PI (*P =* 0.0003) where IL-1β and TNF-α administration resulted in greater bodyweight loss when compared to controls (IL-1β: P_adj_ = 0.0037; TNF-α: P_adj_ = 0.0002, Fig. 1b). The 24 h timepoint reflected the most severe drop in bodyweight with a gradual return to baseline, which was reached at 72 h (Supplementary Figure 1f). Food consumption was also altered across groups (*P =* 0.0003) and *post hoc* analysis revealed that both IL-1β- and TNF-α-treated mice consumed less food in the 24 h PI (IL-1β: P_adj_ = 0.0005, TNF-α: P_adj_ = 0.0004, Fig. 1c). Food intake returned to baseline at 48 h PI (Supplementary Figure 1g). At 24 h PI, no statistically significant effects were observed in water intake (*P =* 0.6227, Fig. 1d) or across the 144 h PI (*P =* 0.4359, Supplementary Figure 1h). Similarly, there were no differences between control and treatment groups for rectal body temperature at 2 h PI (*P =* 0.6679, Supplementary Figure 1i) or across the 144 h PI (*P =* 0.7236, Supplementary Figure 1j).

Despite evidence that IL-6 is essential for the sickness response [88–91], we were unable to demonstrate this with IP administration of IL-6 as no differences were detected at 24 h PI for locomotor activity (*P =* 0.7421, Fig. 1e), bodyweight (*P =* 0.2601, Fig. 1f), or food intake (*P =* 0.8519, Fig. 1g). While there was a trend for decreased water consumption following IL-6 administration, this did not reach statistical significance (*P =* 0.0591, Fig. 1h). Groups did not differ in baseline bodyweight (*P =* 0.6896, Supplementary Figure 1k) and although bodyweight trajectory significantly differed across the groups following IL-6 administration (*P* < 0.0001, Supplementary Figure 1l), *post hoc* analysis revealed that this was not an overall treatment effect. Despite IL-6 being a key mediator of fever, there were no differences in rectal body temperature at 2 h PI (*P =* 0.3995, Supplementary Figure 1m) or for the remainder of monitoring (*P =* 0.5856, Supplementary Figure 1n). However, as exogenous administration of IL-6 may require a non-pyrogenic dose of IL-1β [92, 93], we assessed whether the combination of IL-6 and a non-pyrogenic dose of IL-1β could elicit a sickness response. Even with the addition of IL-1β, no significant differences were observed in locomotor activity (*P =* 0.3297, Fig. 1i). While baseline bodyweights did not significantly differ between groups (*P =* 0.1090, Supplementary Figure 1o), a moderate and statistically significant decrease in bodyweight loss was observed 24 h PI (*P =* 0.0012, Fig. 1j) with recovery at 48 h PI (Supplementary Figure 1p). A trend for decreased food (*P =* 0.0536, Fig. 1k) and water (*P =* 0.0522, Fig. 1l) consumption was observed, however, this effect did not reach statistical significance. While the combination of IL-6 and IL-1β did result in a trend for increased rectal body temperature at 2 h PI, this did not reach statistical significance (*P =* 0.0568, Supplementary Figure 1q) and no difference in temperature trajectory was observed (*P =* 0.4512, Supplementary Figure 1r). These results suggest that while IL-6 + IL-1β can elicit a partial sickness response, it has weaker effects which are not as robust as IL-1β or TNF-α. Given the non-existent or weak sickness responses elicited by IL-6 and IL-6 + IL-1β, these cytokines were not used in further experiments.

### Paternal IL-1β and TNF-α administration does not affect basic breeding and litter parameters or maternal care

**Fig. 2:**
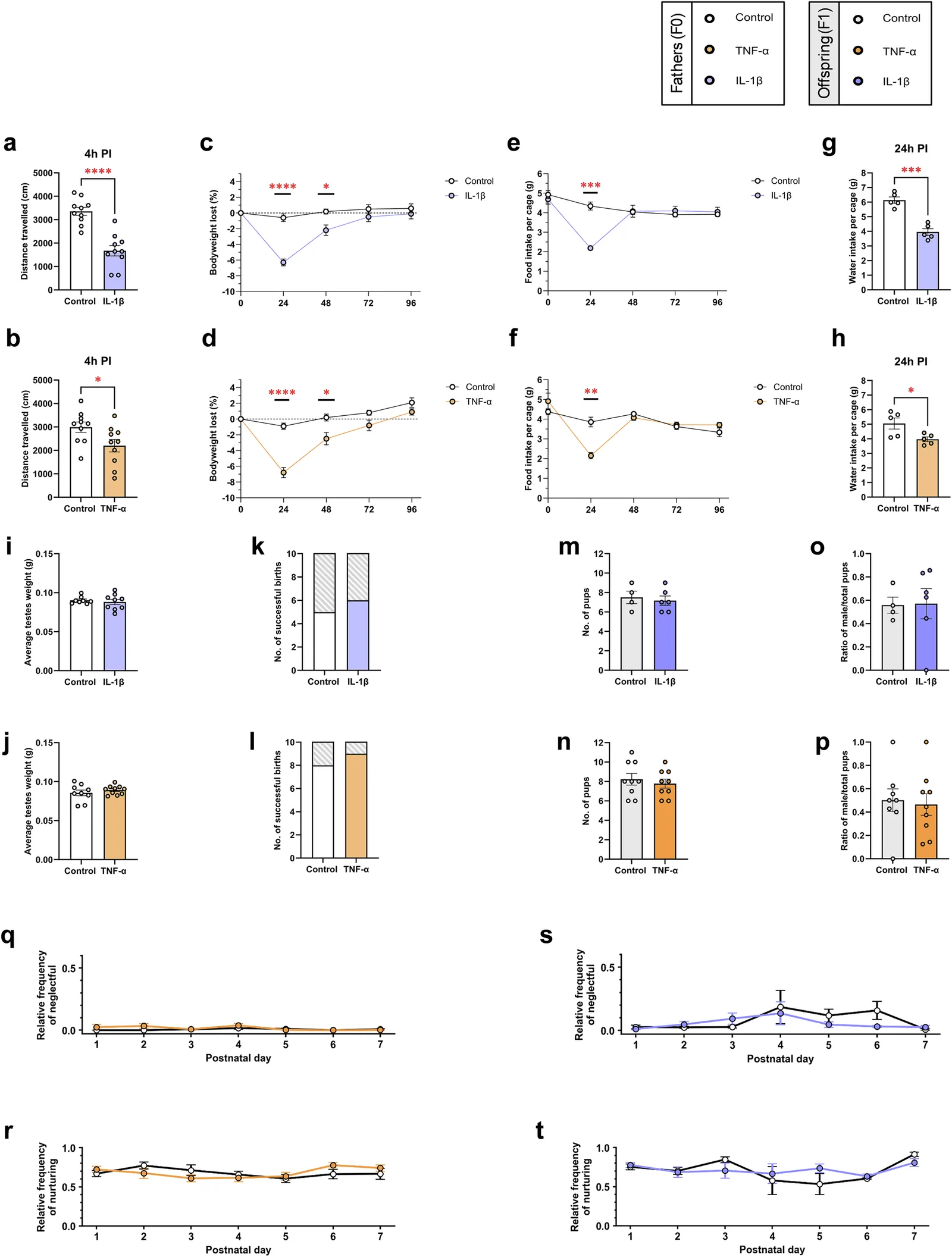
Paternal TNF-α and IL-1β administration and their effects on breeding and litter parameters and maternal care. Male mice were given a single intraperitoneal (IP) injection of IL-1β (50 μg/kg) or TNF-α (250 μg/kg) or vehicle between 8–9 weeks of age and sickness behavior was measured. **(a-b)** Locomotor activity 4 h post-injection (PI) in control, IL-1β, or TNF-α groups. **(c-d)** Bodyweight loss 0–96 h PI in control, IL-1β, or TNF-α groups. **(e-f)** Food intake 0–96 h PI in control, IL-1β, or TNF-α groups. **(g-h)** Water intake 24 h PI in control, IL-1β, or TNF-α groups. **(i-j)** Combined average testes weight of the left and right testes from F0 male mice. **(k-l)** Number of successful births in each cohort out of 10 matings per group. **(m-n)** Number of pups per litter. **(o-p)** Sex ratio of litters. **(q-r)** Relative frequency of nurturing maternal care. **(s-t)** Relative frequency of neglectful maternal care. Individual values and means are presented with error bars representing ± SEM. For paternal cytokine administration and breeding, males *n* = 10 per group; females *n* = 10 per group. For litter parameters and maternal care *n* = 4–10 litters per group. *p < 0.05, **p < 0.01, ***p < 0.001, ****p < 0.0001.

In two separate cohorts, F0 male breeders were administered IP injections of either IL-1β or vehicle, or TNF-α or vehicle. IL-1β- or TNF-α-treated mice showed a robust sickness response across all parameters measured when compared to vehicle-treated mice. For instance, there was a significant reduction in locomotor activity following both IL-1β (*P *< 0.0001, Fig. 2a) and TNF-α (*P =* 0.0334, Fig. 2b) administration. As observed previously, bodyweight was altered in response to cytokine administration with a significant time*paternal treatment interaction effect in the IL-1β group (*P* < 0.0001, Fig. 2c) and TNF-α group (*P *< 0.0001, Fig. 2d). *Post hoc* analysis revealed that cytokine-treated F0 male mice experienced the greatest drop in bodyweight at 24 h PI (IL-1β: *P *< 0.0001; TNF-α: *P* < 0.0001). Partial recovery was achieved at 48 h PI, however both TNF-α and IL-1β-treated males were still underweight compared to controls (IL-1β: *P* < 0.0125; TNF-α: *P* < 0.0358). A gradual return to baseline was observed at 72 h (IL-1β:*P =* 0.6045; TNF-α: *P =* 0.1984). Food intake followed a similar trajectory with a significant interaction effect between time*paternal treatment for IL-1β (*P* < 0.0001, Fig. 2e) and TNF-α (*P* < 0.0001, Fig. 2f), whereby food intake was significantly reduced at 24 h PI (IL-1β: *P* < 0.0005; TNF-α: *P =* 0.0034) and returned to normal at 48 h in both groups (IL-1β: *P* > 0.9999; TNF-α: *P =* 0.7536). Water intake was also significantly reduced at 24 h PI for both IL-1β (*P *< 0.001, Fig. 2g) and TNF-α (*P =* 0.0301, Fig. 2h). These results suggest that the sickness response is transient and most severe at 24 h with a gradual return to baseline, which was reached at 72 h. After a rest period of 4 weeks, male mice were individually mated with naïve wildtype female mice to give rise to F1 offspring. To confirm that the transient sickness response did not impact gross reproductive measures, we assessed breeding and litter parameters. Following the mating period, F0 male tissues were collected. No significant differences were observed in the average testes weight of fathers administered IL-1β (*P =* 0.5632, Fig. 2i) or TNF-α (*P =* 0.3699, Fig. 2j). Similarly, no differences were observed across successful matings between control and paternal treatment groups (Fig. 2k–l), number of pups per litter (IL-1β: *P =* 0.6823; TNF-α: *P =* 0.6836, Fig. 2m, n), or the sex ratio of pups born (IL-1β: *P =* 0.9432; TNF-α: *P =* 0.7898, Fig. 2o, p). The frequency of high-quality maternal care including behavior such as grooming of pups and arch-backed nursing, are known to impact offspring behavior [94, 95]. As such, we measured maternal care over PND 1-7 and found no significant effects of paternal treatment on maternal care across the IL-1β  (neglectful: *P =* 0.510; nurturing: *P =* 0.860, Fig. 2q, r), or TNF-α (neglectful: *P =* 0.555; nurturing: *P =* 0.628, Fig. 2s, t) cohorts. Collectively these results suggest that the administration of IL-1β and TNF-α elicited robust sickness responses without altering gross reproductive measures and maternal care.

### Paternal TNF-α and IL-1β administration differentially alter offspring anxiety-like and stress-coping behavior in a sex-dependent manner

**Fig. 3:**
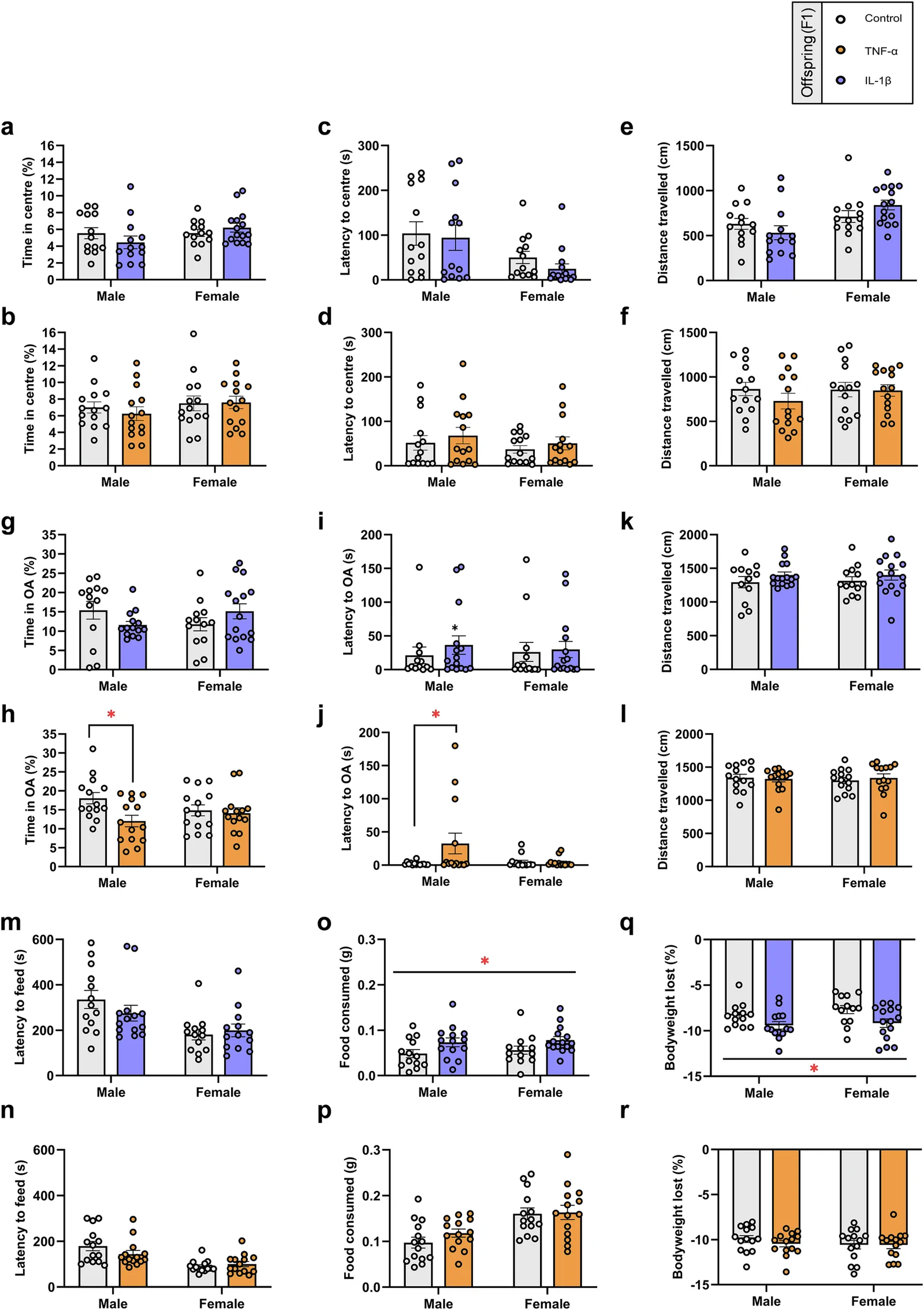
Effects of paternal TNF-α and IL-1β administration on F1 offspring anxiety-like behavior. F1 offspring from IL-1β- or TNF-α-treated fathers underwent a behavioral battery after reaching adulthood at 8 weeks of age. Anxiety-like behavior was assessed. **(a-b)** Time spent in centre (%) of the large open field test (LOF). **(c-d)** Latency to centre in the LOF. **(e-f)** Distance travelled in LOF. **(g-h)** Duration in open arms (OA) of the elevated plus maze (EPM). **(i-j)** Latency to OA in the EPM. **(k-l)** Distance travelled in EPM. **(m-n)** Latency to feed on a chow pellet in the novelty-supressed feeding test (NSFT). **(o-p)** Food consumed immediately after the NSFT trial. **(q-r)** Bodyweight lost following a 24 h fast during the NSFT. Individual values and means are presented with error bars representing ± SEM (*n* = 13–16 per group). *p < 0.05.

At 8 weeks of age, F1 offspring underwent a series of behavioral tests to assess for alterations to anxiety-like, social, stress-coping, and depression-like behaviors. For anxiety-like behavior, both cohorts showed no significant alterations in the LOF regarding proportion of time spent in the centre (IL-1β: *P =* 0.721; TNF-α: *P =* 0.733, Fig. 3a, b), latency to the centre (IL-1β: *P =* 0.218; TNF-α: *P =* 0.307, Fig. 3c, d), or distance travelled (IL-1β: *P =* 0.833; TNF-α: *P =* 0.555, Fig. 3e, f). Similarly, IL-1β offspring did not show significant differences in the time spent in the open arms of the EPM in both females (*P =* 0.210, Fig. 3g) and males (*P =* 0.189, Fig. 3g). In contrast, male offspring from TNF-α-treated fathers spent less time in the open arms of the EPM when compared to their control counterparts (*P =* 0.027, Fig. 3h) while female offspring showed no such difference (*P =* 0.722, Fig. 3h). Only male offspring from TNF-α-treated fathers demonstrated increased latency to enter the open arms (*P *< 0.0001, Fig. 3i, j) with no differences in distance travelled (IL-1β: *P =* 0.180; TNF-α: *P =* 0.766, Fig. 3k, l). Results from the NSFT showed that offspring from neither cohort exhibited any statistically significant differences in latency to feed on the chow pellet (IL-1β: *P =* 0.887; TNF-α: *P =* 0.630, Fig. 3m, n). Interestingly, over the 5 min spent feeding on a chow pellet immediately after the latency trial, offspring from IL-1β-treated fathers and not TNF-α, consumed significantly more food when compared to their respective controls (IL-1β: *P =* 0.039; TNF-α: *P =* 0.369, Fig. 3o, p). Additionally, IL-1β offspring lost significantly more bodyweight (*P =* 0.048, Fig. 3q) following a 24 h fast while TNF-α offspring did not exhibit this phenotype (*P =* 0.521, Fig. 3r). While this presents a potential confound for the interpretation of latency to feed as an anxiety-like behavior, it also suggests alterations to fasting response characterized by heightened levels of weight loss and food consumption post-fast.

**Fig. 4:**
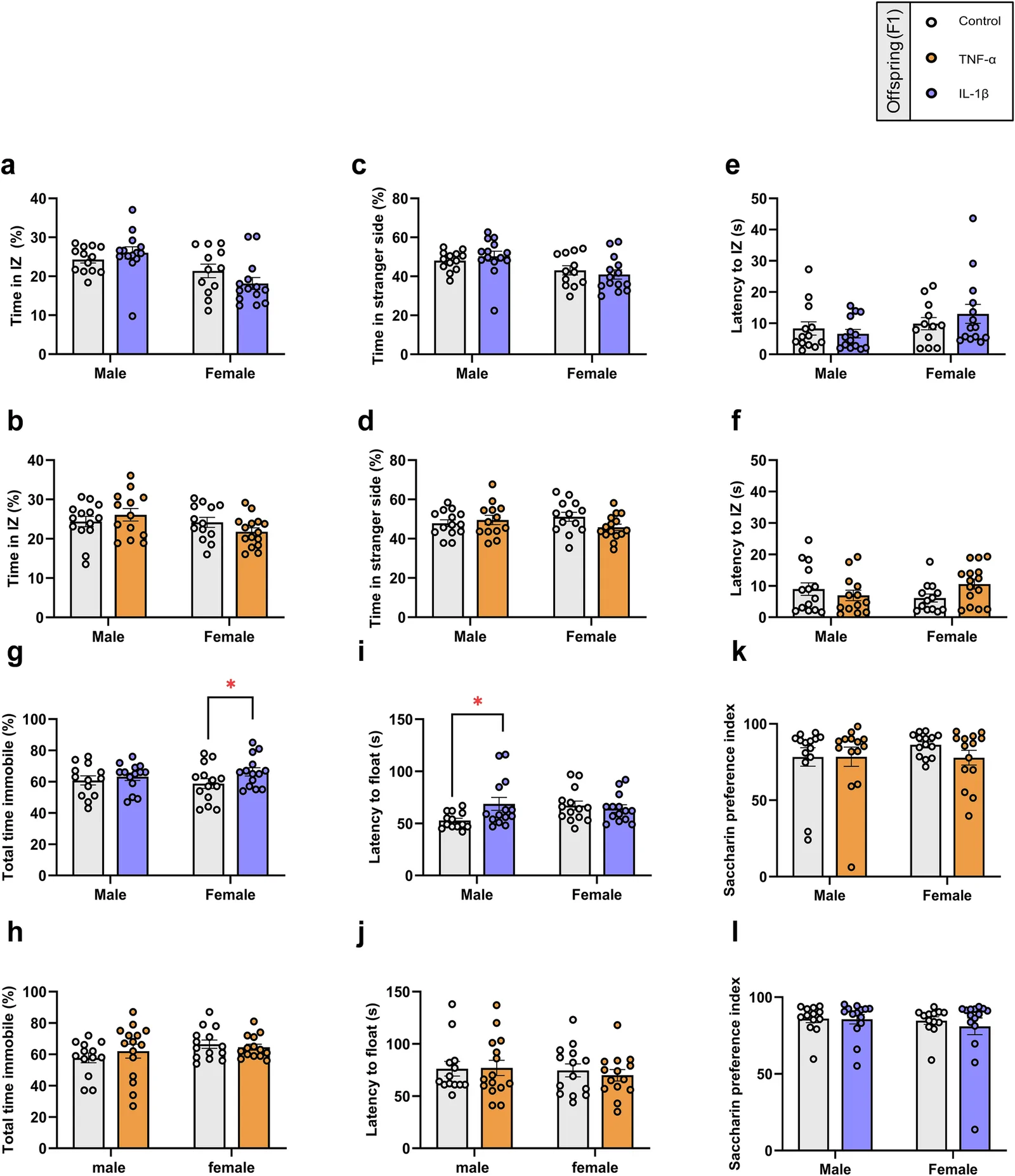
Effects of paternal TNF-α and IL-1β administration on F1 offspring social, stress-coping, and depression-like behavior. F1 offspring from TNF-α- or IL-1β-treated fathers underwent a behavioral battery after reaching adulthood at 8 weeks of age. Sociability, stress-coping, and depression-like behavior were examined. **(a-b)** Time in the interaction zone (IZ) of the social interaction test (SIT). **(c-d)** Time in the stranger side of SIT arena. **(e-f)** Latency to the IZ in SIT. **(g-h)** Time immobile in the Porsolt swim test (PST). **(i-j)** Latency to float in PST. **(k-l)** Saccharin preference index in the saccharin preference test (SPT). Individual values and means are presented with error bars representing ± SEM (*n* = 13-16 per group). *p < 0.05.

Offspring from both IL-1β- and TNF-α-treated fathers showed no significant differences in sociability during the SIT. There were no statistically significant differences in either cohort for the time spent in the interaction zone (IL-1β: *P =* 0.754; TNF-α: *P =* 0.812, Fig. 4a, b), in the side of the arena containing the stranger mouse (IL-1β: *P =* 0.986; TNF-α: *P =* 0.812, Fig. 4c, d), or in the latency to enter the interaction zone with the stranger mouse (IL-1β: *P =* 0.897; TNF-α: *P =* 0.584, Fig. 4e, f). Evaluation of stress-coping behavior demonstrated that IL-1β female offspring spent significantly more time immobile in the PST (*P =* 0.045, Fig. 4g), however, paternal IL-1β did not have the same effect on male offspring (*P =* 0.574, Fig. 4g). Offspring from TNF-α-treated fathers showed no significant differences in time immobile (*P =* 0.690, Fig. 4h). Regarding latency to float, IL-1β male offspring took significantly longer to float (*P =* 0.004, Fig. 4i) while TNF-α offspring showed no such difference when compared to control counterparts (*P =* 0.721, Fig. 4j). For both cohorts, there were no statistically significant differences in anhedonia with similar preferences for saccharin across control and paternal treatment offspring (IL-1β: *P =* 0.579; TNF-α: *P =* 0.447, Fig. 4k, l). Consequently, paternal IL-1β and TNF-α treatment had differential effects on offspring behavior with offspring from TNF-α-treated fathers exhibiting increased anxiety-like behavior and offspring from IL-1β-treated fathers showing altered stress-coping behavior and fasting responses.

### Paternal TNF-α and IL-1β administration do not alter offspring growth trajectory but paternal TNF-α reduces offspring hypothalamus mass in a sex-dependent manner

**Fig. 5:**
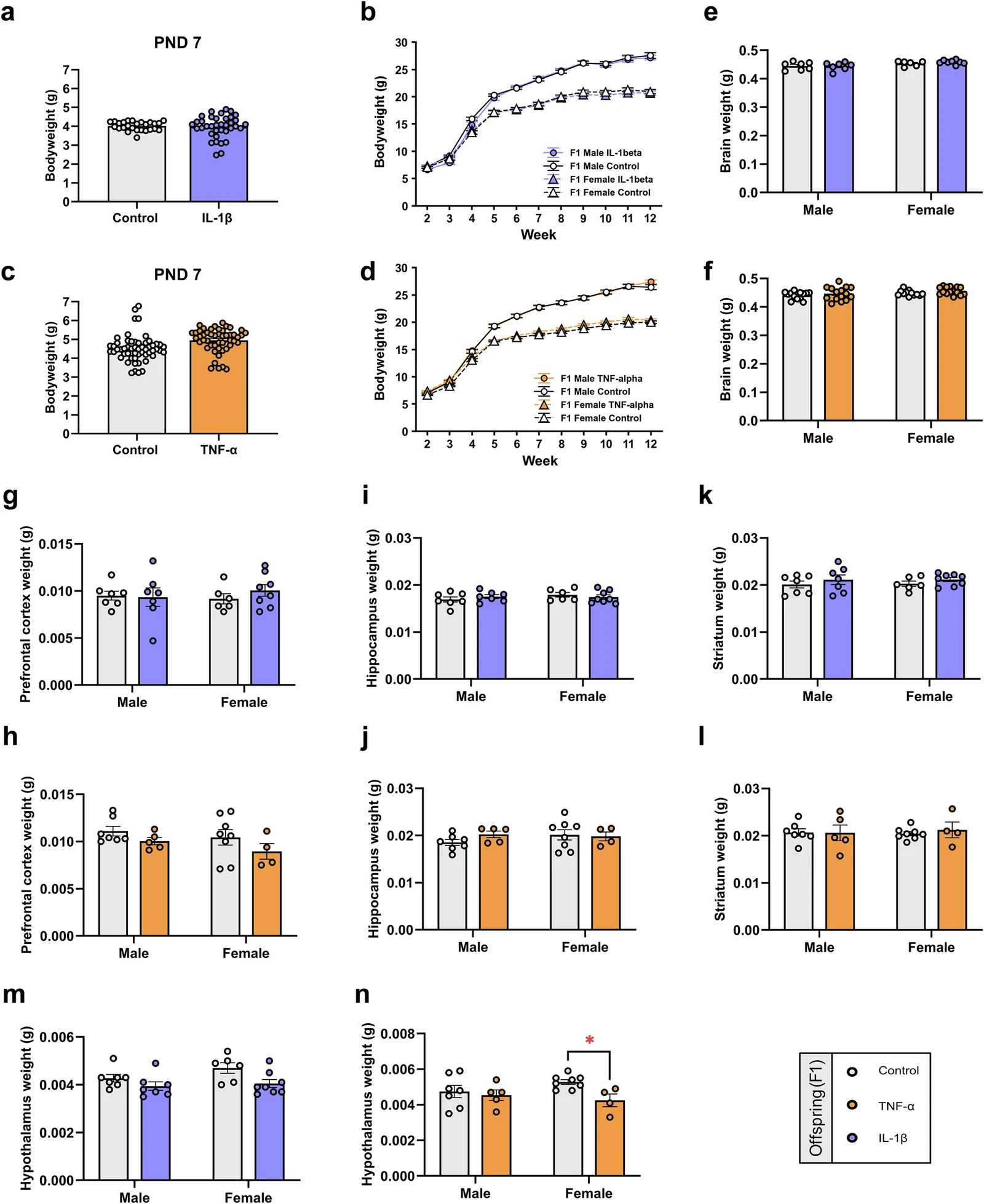
The effects of paternal IL-1β and TNF-α on F1 growth trajectories and brain anatomical measures. Body weight was recorded across the lifetime of F1 mice. **(a)** IL-1β bodyweight at postnatal day (PND) 7 prior to being assigned sex or ID. **(b)** IL-1β bodyweight trajectory from PND 14 (week 2) onwards. **(c)** TNF-α bodyweight at PND 7 prior to being assigned sex or ID. **(d)** TNF-α bodyweight trajectory from PND 14 (week 2) onwards. Bodyweights from week 4 onwards are after post-weaning and mixing of litters. Physiological measures of bodyweight and gross structural changes in brains were assessed for offspring from IL-1β- and TNF-α-treated fathers. **(e-f)** Offspring whole brain mass. **(g-h)** Offspring prefrontal cortex mass. **(i-j)** Offspring hippocampus mass. **(k-l)** Offspring striatum mass. **(m-n)** Offspring hypothalamus mass. Individual values and means are presented with error bars representing ± SEM. (For bodyweight data *n* = 12–16, for brain weights, *n* = 6–16). *p < 0.05.

Previous studies have demonstrated that environmental insults often alter offspring developmental trajectories. Consequently, we monitored offspring bodyweight trajectory weekly across experimentation and assessed food and water consumption post-weaning up until the beginning of behavioural testing. At PND 7 no statistically significant differences were observed in bodyweight across the IL-1β and control groups (*P =* 0.905, Fig. 5a). Although analysis of bodyweight trajectory revealed a significant paternal treatment*sex*time interaction effect (P < 0.0001, Fig. 5b), *post hoc* analysis did not reveal any significant differences between control and paternal treatment for either sex at the time points measured. In the TNF-α cohort, there was no difference in offspring bodyweight at PND 7 (*P =* 0.471, Fig. 5c) however, bodyweight trajectory showed a significant sex*time interaction effect (P < 0.0001, Fig. 5d), indicating expected sex differences in bodyweight with no paternal treatment effects. Consequently, these results indicate that paternal cytokine treatment and the ensuing transient sickness response did not have a significant effect on offspring bodyweight trajectory. In line with these results, IL-1β and TNF-α cohorts showed no significant alterations to food (IL-1β: *P =* 0.105; TNF-α: *P =* 0.213) or water (IL-1β: *P =* 0.826; TNF-α: *P =* 0.860) intake during early life (Supplementary Figure 2a–d).

Following the completion of behavioral tests, offspring were culled for tissue collection. Brain weight measurements were assessed as a gross indicator of brain structure. Paternal cytokine treatment had no statistically significant effects on whole brain mass (IL-1β: *P =* 0.885; TNF-α: *P =* 0.199, Fig. 5e, f). Across the key brain regions investigated, there were no significant differences in the mass of prefrontal cortex (IL-1β: *P =* 0.717; TNF-α: *P =* 0.144, Fig. 5g, h), hippocampus (IL-1β: *P =* 0.894; TNF-α: *P =* 0.555, Fig. 5i, j), or striatum (IL-1β: *P =* 0.429; TNF-α: *P =* 0.758, Fig. 5k, l). While offspring from IL-1β-treated fathers showed a subtle reduction in offspring hypothalamus mass, this effect did not reach statistical significance (*P =* 0.067, Fig. 5m). However, paternal TNF-α treatment did lead to a significant reduction in hypothalamus mass for female offspring (*P =* 0.048, Fig. 5n) but not males (*P =* 0.511, Fig. 5n). These results suggest that IL-1β and TNF-α have limited effects on offspring development trajectories with the only detectable changes occurring in offspring hypothalamuses.

### Paternal sperm small ncRNA profiles are altered in response to administration of IL-1β and TNF-α

Paternal preconceptual exposure to immune activation can alter offspring behavior and physiology via alterations to sperm small non-coding RNA profiles [25–28]. As cytokine treatment with IL-1β and TNF-α elicit a similar sickness response to other PIA models and appears to alter offspring behavior in overlapping ways, we investigated whether this may occur via changes to the small ncRNA profile of paternal epididymal sperm. Specifically, we analyzed sperm within the caudae epididymides as this region contains the mature sperm responsible for fertilising the ova.

**Fig. 6:**
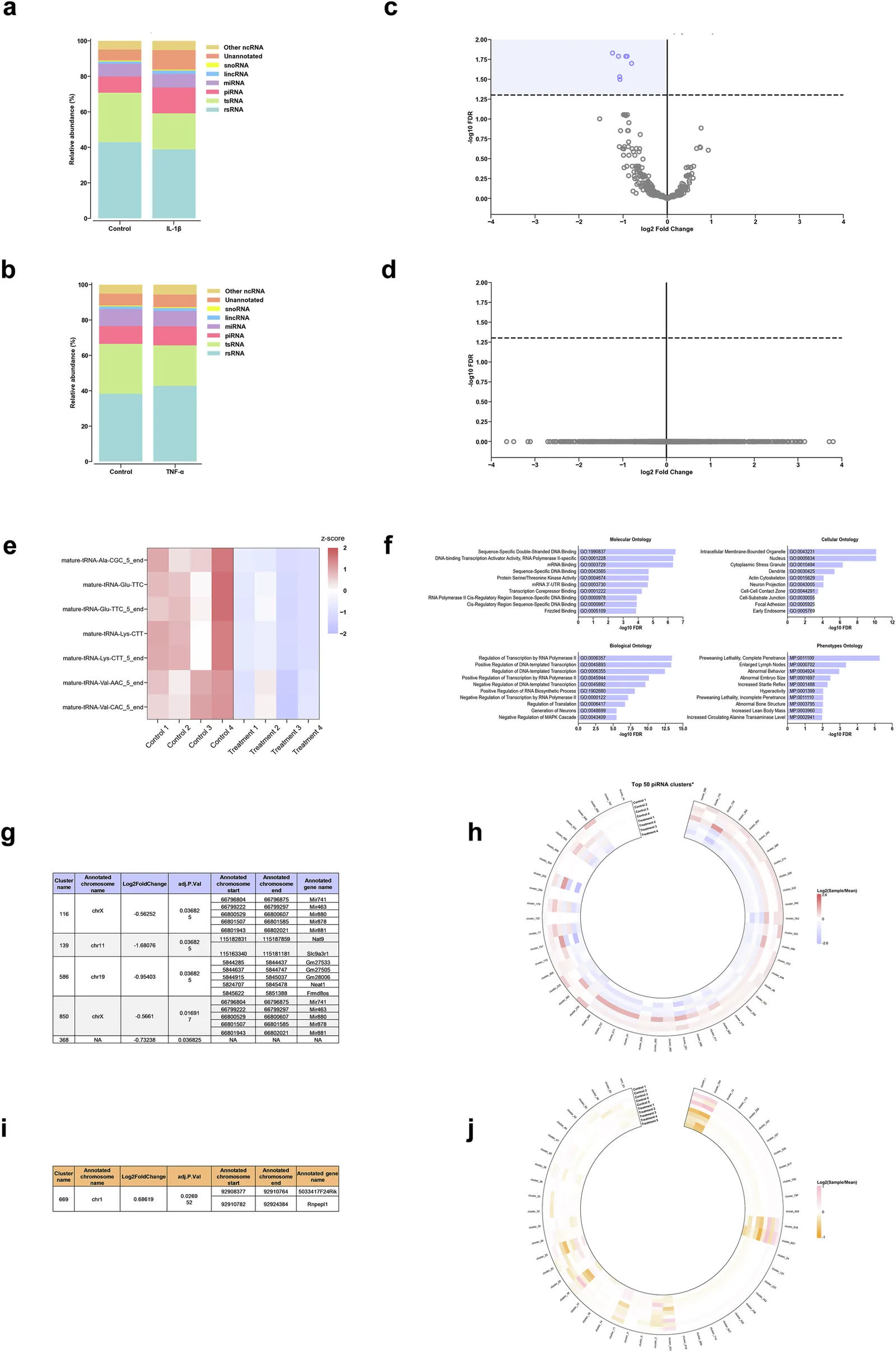
Alterations in paternal sperm small RNA following TNF-α and IL-1β administration. Male mice were given a single IP injection of TNF-α or IL-1β, or vehicle at 8-9 weeks of age. Approximately 5 weeks following cytokine administration, sperm was collected and sequenced for analysis. (**a)** Relative abundance of small ncRNA classes in control vs IL-1β groups **(b)** Relative abundance of small ncRNA classes in control vs TNF-α groups **(c)** Volcano Plot: 7 downregulated tsRNAs in the IL-1β group. Changes are graphed according to log2(fold change) (x-axis) and −log10(FDR) (y-axis). The significance level is set at FDR < 0.05, represented by a horizontal dash line. Downregulated small RNAs in IL-1β are coloured in purple. (**d)** Volcano Plot: no differentially expressed small ncRNAs detected in the TNF-α group. **(e)** Heatmap: expression levels of the 7 downregulated tsRNAs in the IL-1β group, graphed with normalized counts **(f)** Gene ontology analysis of predicted gene targets reveals pathways associated with biological, cellular, molecular, and knock-out mouse project phenotype ontology ranked by P-value. **(g)** Differentially expressed piRNA clusters and their annotations, where possible, found in sperm of IL-1β-treated fathers. **(h)** Heatmap: expression levels of the top 50 most significant piRNA clusters in the IL-1β group. **(i)** Differentially expressed piRNA clusters and their annotations found in sperm of TNF-α-treated fathers. **(j)** Heatmap: expression levels of the top 50 most significant piRNA clusters in the TNF-α group.

The sperm small ncRNA profile can be characterized by assessing the total number of reads which map to each subclass [96]. As such, we evaluated the population of small nucleolar RNA (snoRNA), long intergenic noncoding RNA (lincRNA), miRNA, piRNA, tsRNA, and rsRNA as a percentage of total reads to provide a snapshot of the small ncRNA composition [22, 38, 97] (Fig. 6a, b). Interestingly, the tsRNA population in sperm was significantly reduced in fathers treated with IL-1β (*P*_*adj*_ = 0.000891) with no such difference in fathers treated with TNF-α (*P*_*adj*_ = 0.778783). Analysis of individual small ncRNAs found no significant differences in expression of snoRNA, lincRNA, miRNA, or rsRNA in response to either cytokine. Paternal IL-1β treatment did however result in the downregulation of 5 different tsRNAs: 5’ fragment of tRNA-Glu-TTC, 5’ fragment of tRNA-Lys-CTT, 5’ fragment of tRNA-Ala-CGC, 5’ fragment of tRNA-Val-CAC, and 5’ fragment of tRNA-Val-AAC (FDR < 0.05) (Fig. 6c). Additionally, the combined counts of tsRNAs derived from 5’ end, 3’ end, 3’CCA end, and internal locations were downregulated for 'mature-tRNA-Glu-TTC’ and ‘mature-tRNA-Lys-CTT’ (FDR < 0.05) (Fig. 6c). A similar log2fold change was observed across these tsRNAs, ranging from −0.81 to −1.24 (Fig. 6e) and gene ontology analysis revealed that their targets are involved in processes including DNA binding, regulation of transcription and translation, and KOMP2 phenotypes for abnormal behavior and hyperactivity (Fig. 6f). Surprisingly, TNF-α-treated fathers showed no significant alterations in individual sperm small ncRNA of fathers (Fig. 6d).

As piRNAs functionally operate in clusters, identification of individual differentially expressed piRNAs may not adequately reflect their functional effects on the developing embryo. Therefore, we conducted piRNA cluster analysis. Results revealed 5 significantly downregulated piRNA clusters in sperm from IL-1β-treated fathers: cluster 116 (*P*_*adj*_ = 0.036825); cluster 139 (*P*_*adj*_ = 0.036825); cluster 368 (*P*_*adj*_= 0.036825); cluster 586 (*P*_*adj*_ = 0.016917); and cluster 850 (*P*_*adj*_ = 0.036825). As evidenced in Fig. 6g-h, cluster 139 showed the greatest log2fold change of −1.68, while the other clusters showed a relatively lower change in expression. Cluster 139 was found to align to chr11, while cluster 586 aligned to chr19. Interestingly, the piRNA clusters 116 and 850 were both found to align to chrX, in regions near miRNA genes. A single piRNA cluster, 699, was altered in response to paternal TNF-α treatment (*P*_*adj*_ = 0.02695) and aligned to chr1 (Fig. 6i-j). As such, IL-1β and TNF-α administration alters tsRNAs and/or piRNAs in paternal sperm with IL-1β causing a greater shift in the sperm small ncRNA profile.

## Discussion

Although PIA is known to alter offspring phenotype [25–28], the responsible component of the immune response remains unknown. In this study, we exogenously administered IL-1β or TNF-α in male mice, at doses which elicited significant sickness responses, and examined effects on sperm small ncRNA profile and subsequent offspring phenotype. We found that paternal TNF-α significantly increased anxiety-like behavior in male offspring and paternal IL-1β led to a significant increase in passive stress-coping behavior in female offspring. Additionally, male and female offspring from IL-1β-treated fathers exhibited altered fasting responses with heightened bodyweight loss and increased food consumption. Alongside these findings, sperm small ncRNA profiles were altered in sires following IL-1β treatment, with downregulation of several tsRNAs and piRNA clusters. In contrast, TNF-α treatment only downregulated a single piRNA cluster. Although previous studies have explored how cytokines alter offspring phenotypes in MIA models [63–68], our study is the first to explore the role of cytokines in the context of PIA. Here we present the first evidence supporting the contribution of cytokine activity to altered F0 sperm epigenome and F1 phenotypes which partially recapitulate the effects of other published PIA models.

Similar to the effects of poly I:C, LPS, and SARS-CoV-2 [26–28], exogenous administration of IL-1β and TNF-α elicited a robust sickness response in males characterized by bodyweight loss, reduced food intake, and reduced water intake. Differential effects were also observed, as IL-1β and TNF-α reduced locomotor activity in contrast to poly I:C [26]. Additionally, while LPS had effects on rectal body temperature [27], this was not observed in the present study. This suggests that while the cytokine profiles induced by poly I:C, LPS, IL-1β, and TNF-α overlap, there are also distinctions which modify the sickness response. This is not unexpected, as LPS, poly I:C, IL-1β, and TNF-α activate signaling pathways that elicit different cytokines at varying levels [43–45].

Previous studies suggest that factors in paternal pre-conceptual environment often alter anxiety-like and depression-like behaviors in a sex-dependent manner [23, 25–28, 98]. The present study demonstrated a similar phenomenon whereby male but not female offspring from TNF-α-treated fathers spent significantly less time in the open arms of the EPM, indicating increased anxiety-like behavior. Although offspring did not show alterations in LOF, divergence between these anxiety tests is not uncommon and may occur due to the heightened anxiogenic environment in the EPM [99]. Furthermore, paternal IL-1β had a female-specific effect, increasing time immobile during the PST. This phenotype has been observed in other models of paternal epigenetic inheritance [23, 26, 98] and while it has been interpreted as depression-like behavior, it is more likely to reflect differences in stress-coping strategies (active or passive) when presented with an acute stressor [72, 73]. This may explain the absence of depression-like behavior in other tests such as the SPT. Importantly, male-specific increases in anxiety-like behavior and altered stress-coping behavior in offspring have been observed across paternal SARS-CoV-2 and poly I:C models respectively, where both TNF-α and IL-1β are known to be elevated [26, 28].

In response to paternal IL-1β, offspring also showed altered fasting responses characterized by heightened bodyweight loss and increased food intake. Of note, this effect has been observed in several other PIA mouse models, including those involving paternal poly I:C (viral mimetic) or SARS-CoV-2 (COVID-19) exposures [26, 28]. A sex-specific effect was also observed in a paternal LPS (bacterial mimetic) model where female offspring from LPS-treated fathers showed greater bodyweight loss and increased food consumption post-fast [27]. As such, there is consistent evidence for altered fasting responses in all PIA models where fasting has been conducted. Although poly I:C and SARS-CoV-2 mouse models of PIA have found differences in offspring bodyweight trajectory [26, 28], the present study saw no differences across either cohort. Furthermore, food and water consumption did not differ during early life. Therefore, it is unclear whether the altered fasting responses in our present study reflect a metabolic-like effect. However, previous research suggests that paternal inflammation can induce metabolic disorders in offspring, a process that is mediated at the molecular level by angiogenin, which cleaves mature tRNAs to produce tsRNAs during various stress responses [100].

Interestingly, offspring behavioral and physiological changes were accompanied by an overall trend for decreased hypothalamus weights in offspring from IL-1β-treated fathers and a statistically significant decrease in hypothalamus weights of female offspring from TNF-α-treated fathers. It is unclear how reduced hypothalamus mass impacts offspring phenotype, however, the hypothalamus has known functions in metabolism including appetite and feeding behavior [101, 102]. Furthermore, it is implicated in stress, depression, and anxiety via the hypothalamus-pituitary-adrenal (HPA) axis. While this could have contributed to paternal IL-1β offspring phenotypes, in paternal TNF-α offspring, the decrease in female hypothalamus weights did not match the male-specific behavioral changes. Given the findings of the present study, further research could examine the role of the hypothalamus in altering offspring phenotypes in PIA models.

The paternal IL-1β and TNF-α offspring phenotypes most closely resemble the effects of PIA via viral infection or viral mimetics. Paternal administration of IL-1β (the present study), poly I:C (a viral mimetic), or SARS-CoV-2 (modeling COVID-19), have overlapping effects on offspring, including altered stress-coping and fasting responses [26]. Similarly, paternal TNF-α has some overlap with paternal SARS-CoV-2 infection (modeling COVID-19) as they both alter anxiety-like behavior in offspring, most prominently in males. Surprisingly, paternal cytokine administration showed no overlap in offspring phenotype with the bacterial mimetic (LPS) model of PIA [27]. This suggests that the administration of IL-1β or TNF-α and the subsequent immune profile likely resembles viral infection. However, this does not exclude the possibility that IL-1β and TNF-α are contributing to altered offspring phenotype in paternal LPS models, as different doses and/or combinations of cytokines may cause different epigenetic changes. Nonetheless, given the doses in the present study, IL-1β and TNF-α altered offspring phenotypes similarly to viral, but not bacterial, PIA mouse models.

Paternal pre-conceptual environmental insults may alter offspring phenotypes via various epigenetic mechanisms including alterations to sperm small ncRNAs. Sperm-delivered RNAs can modulate gene expression during early embryonic development and thus impact offspring phenotypes [30, 31]. In the present study, both cytokines induced differential expression of sperm small ncRNAs, all of which were downregulated, similar to PIA via poly I:C [26]. Within the IL-1β cohort, 7 different tsRNAs were significantly downregulated. The tsRNAs include tRNA-derived fragments (tRFs) and tRNA-derived stress-induced small RNAs (tiRNAs), which are generated from the cleavage of tRNA and play key roles in gene regulation by modulating mRNA stability and translation in a similar manner to miRNA [103, 104]. Although their known functions are still emerging, tsRNAs are considered essential regulators of spermatogenesis, pre-implantation, and cellular stress [105–108]. Consistent with the role of tsRNAs, several of the tsRNAs downregulated in this study have known involvement in immunity. The 5’ fragment of tRNA-Ala-CGC induces IL-8 production [109] while the blocking of 5’ fragment of tRNA-Lys-CTT, along with the 5’ fragment of tRNA-Glu-CTC, can lead to the suppression of viral replication [110, 111]. One of the downregulated tsRNAs in response to IL-1β was 5′ fragment of tRNA-Val-CAC. Interestingly, the 5′ fragment of tRNA-Val-CAC-2 is known to be highly abundant in mature mouse sperm, and can be delivered to sperm via extracellular vesicles [38]. Recently, this tsRNA was found to influence gene expression in the 2-cell stage with its inhibition altering embryonic pre-implantation [108]. These effects could be similar for 5’ fragment of tRNA-Val-AAC, as the sequences are largely indistinguishable [108]. Furthermore, IL-1β treatment saw the downregulation of the 5’ fragment of tRNA-Glu-TTC which has also been downregulated in the placenta following poly I:C exposure in MIA models [112]. This may suggest that overlapping downstream signaling from both poly I:C and cytokine exposure is altering tsRNAs in a similar way. However, the 5’ fragment of tRNA-Glu-TTC being downregulated in different tissues may have distinct effects on the developing embryo. Evidence of cytokines downregulating these tsRNAs, and their connections to immunity, pre-implantation and MIA, further supports how cytokines can alter sperm small ncRNAs in a manner which impacts early embryonic development.

In response to both IL-1β and TNF-α, there was downregulation of piRNA clusters in paternal sperm. In male germ cells, piRNAs are predominantly derived from piRNA clusters, specialized genomic loci that serve as primary sources of single-stranded precursor transcripts [113]. These precursors are processed through the primary biogenesis pathway where they are cleaved, loaded onto PIWI proteins and processed via trimming and methylation. The secondary ‘ping-pong’ pathway amplifies silencing through target-guided cleavage. The piRNAs originating from these clusters can be crucial for transposon repression, ensuring genomic integrity, germ cell maturation, and male fertility [114, 115]. Supporting this, several of the piRNA clusters downregulated in the present study targeted genes involved in reproductive systems, spermatogenesis, and brain development [116–118], along with clusters 116 and 850 targeting the same miRNA genes on chromosome X. Interestingly, piRNA cluster 586 targeted the long non-coding RNA gene *Neat1,* which is involved in paraspeckle formation and can decrease translation of certain mRNAs [119]. Knockdown of *Neat1* expression can disrupt paraspeckle formation during the 4-cell stage and subsequently impact development at the 16-cell stage. Furthermore, knockout models have demonstrated maladaptive behavioral responses to stress, including a panic escape response, and alterations to stress-induced neuronal activity [120]. Downregulation of piRNA cluster 586 could alter *Neat1* expression, which may explain the altered stress-coping behavior observed in female offspring or the increased latency to float in male offspring during the PST. However, future studies are required to elucidate how the downregulation of piRNA cluster 586 influences *Neat1* expression, and the impacts this has on offspring phenotype.

Surprisingly, paternal TNF-α only resulted in the differential expression of a single downregulated piRNA cluster on chromosome 1, however, its target gene functions are unclear. Given the lack of notable differences in small ncRNAs in the sperm of fathers injected with TNF-α, small ncRNAs are unlikely to be driving all aspects of the offspring phenotypic changes observed. Instead, other epigenetic mechanisms involving DNA methylation, histone modifications, and long non-coding RNAs, may contribute more to the alterations in the anxiety-like behavior of male offspring. This may also explain why in a paternal SARS-CoV-2 (COVID-19) model, where TNF-α is an important component of the immune response, micro-injection of sperm RNA enriched for small RNA was only able to weakly recapitulate anxiety-like behavior, most prominently in males [28].

Despite no observed overlap in the small ncRNAs altered by cytokines and previous models of PIA, there may be overlap in other epigenetic mechanisms that were not investigated here. Furthermore, the present study uses more recent and improved pipelines specifically designed for small RNA sequencing analysis [74] which may explain the lack of overlapping alterations to small ncRNAs with other PIA models. Additionally, while the present study controlled for somatic cell contamination in sperm samples, previous PIA models have not, which may also be contributing to these differences [26–28].  Although the present study uses updated methodologies, in these PIA models, and others, the seminal plasma has not been investigated and could be contributing to intergenerational effects, as seminal plasma alone can alter offspring health [121, 122]. Overall, the sperm small ncRNA analyses demonstrates downregulation of several ncRNAs involved in or associated with immunity, reproductive processes, and embryonic development. Such alterations could occur during epididymal transit when the small ncRNA profile undergoes extensive remodelling [38, 40, 41]. During this process, soma-germline communication occurs whereby small ncRNA from epididymal soma are delivered to maturing sperm by epididymosomes. This has been evidenced in a number of studies which have found that mature sperm contain epididymis-derived RNAs, and that environmental insults can similarly alter small ncRNA in both epididymosomes and sperm [41, 123, 124]. As such, it is likely that elevated cytokine levels alter sperm small ncRNA via changes to the RNA cargo of epididymosomes. While the mechanisms through which pro-inflammatory cytokines alter small ncRNA remains unclear, previous studies suggest that cytokines such as IL-1β and TNF-α can induce excessive production of reactive oxygen species (ROS) in various tissues and cell types [125–129]. A similar effect is likely in sperm or reproductive tissue as inflammation and associated cytokines levels are strongly linked with ROS production which has been suggested as a source of male infertility [130, 131]. This occurs as ROS leads to oxidative stress and subsequent DNA damage, along with impacts on epigenetic processes, such as small ncRNA [126, 128, 132]. Even small changes in small non-coding RNA during early embryogenesis may impact offspring development via altered ‘transcriptional cascades’ which can amplify neuronal dysfunction [30]. As such, cytokine induced sperm epigenetic changes in the present study may be contributing to offspring phenotypic changes which affect brain and behavior.

Although paternal cytokine administration has clear effects on sperm small ncRNA and subsequent offspring phenotypes, this study does not explore which cytokine drives this phenomenon as cytokines work in cascades wherein the synthesis and release of one, triggers the production of several others. IL-1β and TNF-α have been shown to have a reciprocal relationship in the brain, where the production of IL-1β induces TNF-α synthesis, and vice versa [52]. Conversely, in the blood it is thought to act unidirectionally with TNF-α eliciting IL-1β [133]. Additionally, both IL-1β and TNF-α can stimulate IL-6 production [134]. Consequently, IL-1β could be driving offspring phenotypes with TNF-α administration inducing IL-1β to a lower level, thus leading to weaker behavioral changes than when IL-1β is directly administered. Additionally, it is unknown whether the cytokine profile in the present study matches that of other PIA models. Similarities and differences in the types and levels of cytokines may help explain the overlapping and diverging offspring phenotypes. Furthermore, when applied to humans, it is still unclear whether the same cytokines of IL-1β and TNF-α would be driving changes as human primary cells respond differently to TLR stimulation [135]. Nonetheless, these findings provide the first insights into how paternal cytokine administration can alter offspring phenotype, highlighting a key component of the immune response which contributes to altered offspring behavior and physiology in PIA models [136].

Psychiatric disorders contribute a significant burden of disease at an individual and societal level. Unfortunately, the precise etiology of many common conditions including depression and anxiety remains elusive, posing a major barrier to both prevention and treatment. Here we demonstrate that elevated levels of IL-1β and TNF-α differentially alter offspring behaviors, increasing anxiety-like and stress-coping behavior in a sex-dependent manner. We also show impacts on offspring physiology with alterations to fasting responses and slight decreases in hypothalamus weight. Offspring phenotypes may be partially explained by changes to small non-coding RNAs which could alter early embryonic development, however, other epigenetic mechanisms are also likely  to be involved. These findings closely resemble the effects of PIA via viral pathogens and their mimetics, indicating that IL-1β and TNF-α may play important roles in altering offspring phenotypes in such models. Understanding the role cytokines play in PIA is relevant in understanding the mechanisms that allow paternal infection to impact offspring health. With further research and understanding of how these findings extend to humans, we could inform efforts to prevent and mitigate any potential increases in the prevalence of psychiatric disorders.

## Supplementary information

SuppLegends

Supplementary Figure 1

Supplementary Figure 2

Supplementary Figure 3

Supplement 1

## Data Availability

Code used in this study will be made available upon request.

## Data availability

Sperm small RNA sequencing data is uploaded to NCBI Gene Expression Omnibus (GEO) study #GSE329980.
