# Supplementary material for: Paternal cytokine administration alters sperm small ncRNAs and offspring brain and behavior
Source: Mol Psychiatry. 2026 Jun 19;31(11):6183–201. doi: 10.1038/s41380-026-03674-5 (PMC13668724; doi:10.1038/s41380-026-03674-5)
Supplement: Supplementary file 1 [file 41380_2026_3674_MOESM1_ESM.docx]

**Supplementary Figure Legends**

**Supplementary Figure 1. Additional measures of sickness responses following a single dose of cytokines.** Male mice at 8-9 weeks of age received a single IP injection IL-6 or PBS with or without BSA. In a separate cohort, male mice were administered IL-1β, or TNF-α, or vehicle. In another cohort male mice were administered vehicle or IL-6 + IL-1β. **(a)** Locomotor activity 4 hours PI of IL-6 or PBS with or without BSA. **(b)** Bodyweight 24 hours PI of IL-6 or PBS with or without BSA. **(c)** Food intake 24 hours PI of IL-6 or PBS with or without BSA**. (d)** Water intake 24 hours PI of IL-6 or PBS with or without BSA**. (e)** Baseline bodyweight in control, TNF-α, or IL-1β groups. **(f)** Bodyweight trajectory up to 144 hours PI in control, TNF-α, or IL-1β groups. **(g)** Food intake up to 144 hours PI in control, TNF-α, or IL-1β groups. **(h)** Water intake up to 144 hours PI in control, TNF-α, or IL-1β groups. **(i)** Temperature change at 2 hours PI in control, TNF-α, or IL-1β groups. **(j)** Temperature change up to 144 hours PI in control, TNF-α, or IL-1β groups. **(k)** Baseline bodyweight in IL-6 or PBS groups with or without BSA. **(l)** Bodyweight trajectory up to 144 hours PI in IL-6 or PBS groups with or without BSA. **(m)** Temperature change at 2 hours PI in IL-6 or PBS groups with or without BSA. **(n)** Temperature change up to 144 hours PI in IL-6 or PBS groups with or without BSA. **(o)** Baseline bodyweight in control or IL-6 + IL-1β groups. **(p)** Bodyweight trajectory up to 144 hours PI in control or IL-6 + IL-1β groups. **(q)** Temperature change at 2 hours PI in control or IL-6 + IL-1β groups. **(r)** Temperature change up to 144 hours PI in control or IL-6 + IL-1β groups. Bars and lines represent mean, with error bars representing ± SEM (males *n* = 6-10 per group), **p* < 0.05.

**Supplementary Figure 2. Food and water intake trajectory for juvenile F1 offspring.** F1 offspring from IL-1β- or TNF-α-treated fathers were weighed weekly along with their food and water intake following weaning. **(A)** Water consumed per cage in offspring from TNF-α-treated fathers **(B)** Water consumed per cage in offspring from IL-1β-treated fathers **(C)** Food consumed per cage in offspring from TNF-α-treated fathers **(D)** Food consumed per cage in offspring from IL-1β-treated fathers.

**Supplementary Figure 3. Additional information for differentially expressed piRNA clusters.** Male mice were given a single IP injection of IL-1β, TNF-α, or vehicle at 8-9 weeks of age. Approximately 5 weeks following cytokine administration, sperm was collected and sequenced for analysis. **(A)** Relative abundance of different small ncRNA classes in sperm from control vs IL-1β-treated fathers **(B)** Relative abundance of different small ncRNA classes in sperm from control vs TNF-α-treated fathers **(C)** Additional statistical details for differentially expressed tsRNAs in sperm from IL-1β-treated fathers (**D)** Differentially expressed piRNA clusters in sperm with additional statistical details for control vs. IL-1β-treated fathers **(E)** Differentially expressed piRNA clusters in sperm with additional statistical details in sperm of control vs. TNF-α-treated fathers.
